# Screening of Feral and Wood Pigeons for Viruses Harbouring a Conserved Mobile Viral Element: Characterization of Novel Astroviruses and Picornaviruses

**DOI:** 10.1371/journal.pone.0025964

**Published:** 2011-10-17

**Authors:** Tone Kofstad, Christine M. Jonassen

**Affiliations:** 1 Department of Laboratory Services, National Veterinary Institute, Oslo, Norway; 2 Division for Diagnostics and Technology, Akershus University Hospital, Lørenskog, Norway; University of Hong Kong, Hong Kong

## Abstract

A highly conserved RNA-motif of yet unknown function, called stem-loop-2-like motif (s2m), has been identified in the 3′ end of the genomes of viruses belonging to different RNA virus families which infect a broad range of mammal and bird species, including *Astroviridae*, *Picornaviridae*, *Coronaviridae* and *Caliciviridae*. Since s2m is such an extremely conserved motif, it is an ideal target for screening for viruses harbouring it. In this study, we have detected and characterized novel viruses harbouring this motif in pigeons by using a s2m-specific amplification. 84% and 67% of the samples from feral pigeons and wood pigeons, respectively, were found to contain a virus harbouring s2m. Four novel viruses were identified and characterized. Two of the new viruses belong to the genus *Avastrovirus* in the *Astroviridae* family. We propose two novel species to be included in this genus, *Feral pigeon astrovirus* and *Wood pigeon astrovirus*. Two other novel viruses, *Pigeon picornavirus A* and *Pigeon picornavirus B*, belong to the *Picornaviridae* family, presumably to the genus *Sapelovirus*. Both of the novel picornaviruses harboured two adjacent s2m, called (s2m)_2_, suggesting a possible increased functional effect of s2m when present in two copies.

## Introduction

An RNA motif, called stem-loop-2-like motif (s2m), has been identified in the 3′ end of the genomes of viruses belonging to different RNA virus families which infect a broad range of mammal and bird species [1]. So far, s2m has been found in species of the following virus families; *Astroviridae*, *Picornaviridae*, *Coronaviridae*, and *Caliciviridae*. All avastroviruses identified to date, except *Turkey astrovirus 2* (TAstV-2) harbour s2m. In addition, several species among mamastroviruses, with the exception of *Bottlenose dolphin astrovirus 1*, and the branch comprising, among others, *Human astrovirus MLB1*, rat astroviruses and a few bat astroviruses [2]–[6], harbour this motif.On the other hand, only few picornaviruses have been identified to date harbouring s2m; viruses in the genus *Erbovirus* (*Equine rhinitis B virus 1* and *2*) causing a mild respiratory infection in horses [7], and more recently in viruses in the genus *Paraturdivirus*, which is proposed as a novel genus in the *Picornaviridae* family [8]. The viruses in this genus (*Turdivirus 2* and *3*) are found to infect wild birds in Hong Kong. In the *Coronaviridae* family, s2m has been found in all members of the genus *Gammacoronavirus*, that all infect birds, [9], and in the *severe acute respiratory syndrome (SARS)-related coronavirus* that consist of SARS coronaviruses from the human outbreak of 2003, and from bats, within the *Betacoronavirus* genus [10]. In addition, s2m has also been found in *Canine norovirus*, in two isolates from Italy [11] and one from Portugal [12]. The two noroviruses from Italy are assigned to genogroup IV, subtype 2, while the norovirus from Portugal are still unassigned. Noroviruses belong to the family *Caliciviridae*, which has a genome organization similar to astrovirus, and are regarded as a major cause of epidemic, nonbacterial gastroenteritis worldwide in humans at all ages [13]–[15].

In these very different viruses, the RNA-motif is highly conserved. The primary structure of s2m consists of 43 nucleotides, and the secondary structure is predicted to form a basal stem of 6–8 base pairs and a 31 nucleotide loop region http://rfam.sanger.ac.uk/family?entry=RF00164#tabview=tab0. About 75% of the s2m sequence is absolutely invariant between viral species. The nucleotides in the loop region show less variation than the nucleotides in the basal stem, but stem nucleotide differences are almost always compensated by covariations to maintain base pairing [16]. Because RNA viruses have a high mutation rate, this suggests that s2m has a very important function in the viruses carrying it.

X-ray crystallography of SARS coronavirus s2m RNA has shown that it has a unique secondary and tertiary structure with a sharp 90° kink of the RNA helix axis perpendicular to the main helix axis [17]. Comparison of the global fold of the SARS s2m RNA to known RNA tertiary structures revealed that the backbone fold of the s2m RNA has a structure corresponding to the 530 loop of 16S rRNA. This loop in 16S rRNA, and proteins that bind to it, are known to be involved in translation initiation. It has been proposed that s2m might bind to one or more factors in the host's translational machinery, which is necessary for initiation of the protein synthesis, even if the structure homology is identified towards prokaryotic rather than eukaryotic rRNA. The virus could thus hijack this machinery for its own purpose. In addition it is possible that the Nsp9 protein in SARS coronavirus can bind to s2m, and thereby facilitate viral polymerase RNA transcription, translation or replication. Hence, s2m can be important for both the protein synthesis and the replication.

The high degree of similarity between s2m from non-related virus families indicates that s2m has a common origin. It is unlikely that s2m has coevolved with the rest of the genome of a single common ancestor virus, because this motif is present only in a limited number of viral species within the viral families where it has been found and because of the large evolutionary distance between them. This suggests that s2m has been transferred between different RNA virus by non-homologous recombination. A relatively high frequency of recombination is observed within species of coronavirus and picornavirus [18]–[21], both in cell culture and in nature. Recombination has also been demonstrated to occur within astroviruses [3], [22]–[24]. RNA recombination enables the exchange of genetic material not only between the same or similar viruses, but also between distinctly different viruses. However, this seems very rare as it depends on coinfection of a single cell by two non-related viruses, and in contrast to recombination within viral species, transfer from one virus family to another cannot involve a homologous recombination mechanism [18], [25], [26].

In the subgroups/species of the *Picornaviridae*, *Coronaviridae* and *Caliciviridae* families where s2m is found, either every virus strain harbour s2m or none. Thus s2m must have conferred some immediate advantage to the virus upon acquisition, thus spreading faster than viruses not harbouring the motif. In the *Astroviridae* family, however, it seems that the motif has been lost in TAstV-2, since this is the only species among avastroviruses not harbouring s2m. The presence of s2m in several, but not all, branches of mamastrovirus could either be due to the motif being lost in several branches, or to be acquired independently in different lines of mamastroviruses.

Viral families where s2m has been found, are all positive-sense single-stranded RNA viruses, with a poly(A) tail in the 3′ end of their genome. S2m is located 40–200 nucleotides upstream of the poly(A) tail, either completely or partially in their untranslated region. Since s2m is such an extremely conserved motif, it is an ideal target for identification of the viruses harbouring it. We have earlier described a reverse transcription (RT) polymerase chain reaction (PCR) analysis which should be able to detect all viruses with poly(A)-tailed, positive-sense single-stranded RNA genome that contains s2m [9]. As s2m seems to be functional in many different viruses, it is plausible that there exist more viruses that contain s2m than those that have been found so far, and the aim of this study was to identify and characterize s2m harbouring viruses in a bird population.

Samples from feral pigeons were collected in 2003, as part of a surveillance project for zoonotic diseases in wild birds, such as influenza A virus and Newcastle disease virus, at the National Veterinary Institute, Oslo, Norway [27]. The samples were also investigated for coronavirus [9]. In this study these samples, and in addition a few samples from wood pigeons, were screened for the presence of s2m. In this paper we also report characterization and prevalence of novel viruses infecting feral and wood pigeons.

## Materials and Methods

### Ethics statement

Permission to capture and euthanize pigeons for sampling was given by the Norwegian Directorate for Nature Management (ref: 2003/3992 ARTS-VI-ID). In addition, cloacal and tracheal swabs were sampled by a hunter from 9 wood pigeons shot during hunting season in Akershus, in August 2005.

### Sampling

107 feral pigeons were caught in Oslo between June and September 2003 as previously described [9]. Cloacal and tracheal swabs were taken post-mortem from each bird, and kept at −70°C until analysis. Body weight was measured and routine necropsy carried out by the Section for Wildlife Diseases at the National Veterinary Institute, Oslo, Norway.

The cloacal and tracheal swabs from the 9 wood pigeons were placed in tubes containing virus transport medium, and kept at −20°C until analysis.

### RNA isolation and RT-PCR for s2m screening

RNA was isolated from swabs with QIAamp® Viral RNA mini kit (QIAGEN, Hamburg, Germany) or with NucliSens® easyMAG™ (bioMérieux, Marcy l'Etoile, France) according to the manufacturers' instructions.

To screen for s2m, 41 feral pigeon samples were randomly selected, and RT-PCR was performed using a primer (s2m-p) located in the most conserved core of s2m toward a generic primer for poly(A)-tailed RNA (Oligo(dT)_20_), which will amplify 40–200 nucleotides [28], [29]. The primers used in this study are listed in Table 1. The cloacal and tracheal swabs were pooled together, and analysed with a two-step RT-PCR. cDNA synthesis was performed using SuperScript™ III Reverse Transcriptase (Invitrogen, Carlsbad, CA, USA), with 2.5 µM Oligo(dT)_20_ (or Anchored Oligo(dT)_20_) primer according to the manufacturer's protocol. The RT reaction was performed in a thermocycler at 50°C for 30 min, followed by an inactivation step at 70°C for 15 min.

**Table 1:** Sequence of primers used in this study.

Target	Name	Sequence (5′→3′)	Orientation
(s2m)	S2m-p	CCGAGTASGATCGAGGG *	Forward
(s2m)	AV12	TTTTTTTTTTTTTTTTTTGC	Reverse
(s2m)	Blend	TTTTTTTTTTTTTTTTTTVN	Reverse
Picornavirus	AV12B	TTTTTTTTTTTGCAATGCCC	Reverse
Picornavirus	555F400	GATAACACCAGCTGATAAGGG	Forward
Picornavirus	PIC3300FX	GTTGYRRTHATGGAYGA	Forward
Picornavirus	603-7F2600	AAGCTGGATGTTGACAAGG	Forward
Picornavirus	PicB-5′UTR	CGTGTGGTATAGTCCGCTG	Forward
Astrovirus	ANV-R1	AATGAAAAGCCCACTTTCG	Reverse
Astrovirus	ANV-F210	GAGTAGCATCGAGGGTACAG	Forward
Astrovirus	Astro-YGDD	TTATGGAGATGAYMGGCT	Forward
Astrovirus	594-9F2400	CCCGACTTCTACAGGAAAAT	Forward
Astrovirus	AstroDueR2400	ATTTTCCTGTAGAAGTCGGG	Reverse
Astrovirus	TAPG-L1	TGGTGGTGYTTYCTCAARA **	Forward

*From Jonassen *et al.* 2005 [9].

**From Tang *et al.* 2005 [31].

Five µl cDNA were amplified using HotStarTaq DNA Polymerase Kit (QIAGEN, Hamburg, Germany) in a 50 µl PCR. The primers used were s2m-p (0.5 µM) and Blend (0.25 µM). The concentration of Mg^2+^ in the reaction was 1.5 mM.

The amplification programme consisted of an initial 15 min step at 95°C, followed by 40 cycles with 94°C for 40 s, 55°C for 20 s and 72°C for 40 s. A final elongation step at 72°C for 5 min was performed, followed by chilling to 8°C.

Human astrovirus serotype 8, an s2m harbouring virus, was propagated in Caco-2 cells, and RNA isolated from the cell supernatant was used as a positive control in all RT-PCR set-ups. Negative controls consisted of RNase/DNase-free water.

### Sequencing and sequence analysis of the s2m PCR products

All PCR-products were purified by using QIAquick PCR Purification Kit (QIAGEN, Hamburg, Germany) according to the manufacturer's instruction, with a final elution volume of 30 µl MilliQ-water (Millipore, Billerica, MA, USA). The sequencing reaction was performed by using the ABI PRISM BigDye Terminator Cycle Sequencing Ready Reaction kit v1.1 (Applied Biosystems, Carlsbad, CA, USA) according to manufacturer's instructions, and the samples were analysed on an ABI PRISM® 3130xl Genetic Analyzer (Applied Biosystems, Carlsbad, CA, USA).

Software used for sequence analysis was Sequencher version 4.1.4 (Gene Codes Corporation; http://www.genecodes.com), and FASTA similarity search.

### Rapid amplification of cDNA ends and primer walking

The sequence information obtained between s2m and the poly(A) tail allowed for designing of two partly overlapping specific reverse primers for the newly identified viruses, and further sequence information was obtained using a Rapid Amplification of cDNA Ends (5′ RACE) and primer walking strategy. The PCR products were sequenced using the ABI PRISM BigDye Terminator Cycle Sequencing Ready Reaction kit v3.1. [1], [28], [29].

### Virus-specific RT-PCR for further sequencing

Once sufficient sequence information was obtained to assign the novel viruses to a specific family, an alternative strategy to 5′ RACE and primer walking was used for further viral sequence characterization, consisting of amplifying directly a long PCR product using upstream primers in conserved family-specific motifs, e.g. in the RNA dependent RNA polymerase (RdRp) gene.

A consensus primer (Astro-YGDD) was designed in the YGDD amino acid motif of astrovirus RdRp. This primer was used together with the primer ANV-R1, and the PCR was performed using the BD Advantage™ 2 PCR System (Clontech, Mountain View, CA, USA). The amplification product provided limited sequence information, allowing for the design of two specific primers, 594-9F2400 and AstroDueR2400, that could be used for sequence characterization of the astrovirus capsid gene. In order to obtain the complete capsid sequence, and some of the polymerase gene, TAPG-L1, a primer previously reported by Tang *et al.* [30], was used together with AstroDueR2400 as well.

A similar approach was used to obtain more of the 5′ end of the nonstructural genes of picornavirus. All PCR products were sequenced using the ABI PRISM BigDye Terminator Cycle Sequencing Ready Reaction kit v3.1.

### Sequence analyses

Softwares used for sequence analysis and phylogeny, in addition to Sequencher and FASTA similarity search, were CLUSTALW Multiple Sequence Alignment Program (http://www.ebi.ac.uk), and MEGA version 4 (Tamura, Dudley, Nei and Kumar 2007: http://megasoftware.net). Pairwise percent identity between nucleotide or amino acid sequences was calculated by BioEdit version 5.0.9 (http://jsbrown.mbio.ncsu.edu/BioEdit/bioedit.html). Hypothetical polyprotein cleavage sites in the picornaviruses were determined by the NetPicoRNA server version 1.0 (http://www.cbs.dtu.dk/services/NetPicoRNA). Prediction of RNA secondary structure was carried out using Mfold version 4.6 [31]: http://mfold.bioinfo.rpi.edu).

### RT-PCR for astrovirus and picornavirus screening

When sequence information of the newly identified viruses was sufficient to assign the viruses to known virus families, generic primers were designed in conserved areas of genes and used to amplify and sequence larger fragments (200–500 nucleotides) of the viral genomes. All the 116 pigeon RNA samples were then screened specifically for the presence of the novel viruses with the primers, and all PCR products were then sequenced for further subgrouping.

#### Picornavirus RT-PCR

The samples were analysed with a two-step RT-PCR. cDNA synthesis was performed using SuperScript™ III Reverse Transcriptase (Invitrogen, Carlsbad, CA, USA) according to the manufacturer's protocol, with 0.125 µM AV12 primer. The RT reaction was performed in a thermocycler at 50°C for 45 min, followed by an inactivation step at 70°C for 15 min.

The PCR was performed with 555F400 as sense primer and AV12B as antisense primer. These primers were designed to amplify 493 bp in the 3′ end of picornavirus RNA in pigeons. 2.5 µl cDNA was added to a 25 µl PCR and amplified by using HotStarTaq DNA Polymerase Kit (QIAGEN, Hamburg, Germany). The concentrations of primers in the reaction were 0.5 µM, and the concentration of Mg^2+^ was 1.5 mM. The amplification programme consisted of an initial 15 min step at 95°C, followed by 40 cycles with 94°C for 40 s, 55°C for 20 s and 72°C for 60 s. A final elongation step at 72°C for 5 min was performed, followed by chilling to 8°C.

#### Astrovirus RT-PCR

The samples were analysed with a two-step RT-PCR as described above. ANV-R1 was used as primer in the RT reaction and as antisense primer in the PCR. ANV-F210 was used as sense primer in the PCR. These primers were designed to amplify 210 bp in the 3′ end of astrovirus in pigeons. The same amplification programme as above was used, but with 35 cycles instead of 40.

## Results

### Screening for s2m harbouring viruses

S2m positive samples identified from the initial screening, showed similarity to two distinct virus families, picornaviruses and astroviruses.

Of the 107 samples from feral pigeons analysed for picornavirus, 32 samples were found to be positive for picornavirus. The samples from wood pigeons were all negative. Cloacal and tracheal swabs were pooled together when the samples were tested. When swabs from 26 positive feral pigeons were tested separately, picornavirus was present in both the cloacal swabs and tracheal swabs in three of the birds, whereas picornavirus only was found in the cloacal swabs in the rest of the birds.

All the positive samples were confirmed by sequencing of PCR products. The alignment of sequences showed that the present pigeon picornaviruses cluster into two groups, called *Pigeon picornavirus A* (PiPV-A) and *Pigeon picornavirus B* (PiPV-B). At the nucleotide level, the distance between PiPV-A and PiPV-B was about 40% in this region (493 nucleotides). 10 samples clusters in group PiPV-A, and 21 samples clusters in group PiPV-B. In one of the pigeons we found both groups, PiPV-A in the tracheal swab and PiPV-B in the cloacal swab.

Of the 107 pooled tracheal and cloacal swabs from feral pigeons, 85 were found to be positive for astrovirus, and of the 9 samples from wood pigeons, 6 were positive. All the positive samples were confirmed by sequencing of the astrovirus screening PCR products. Cloacal and tracheal swabs from 8 of the positive feral pigeons and the 6 positive wood pigeons were tested separately. Astrovirus was present in all tested cloacal swabs; in addition astrovirus were present in tracheal swabs from 6 of the birds (2 wood pigeons and 4 feral pigeons).

27 feral pigeons were shown to be infected with both picornavirus and astrovirus. Earlier, the samples from feral pigeons have been tested by RT-PCR for coronavirus [9], and two of these samples were found to be positive for coronavirus. One of these was also positive for both picornavirus (PiPV-B) and astrovirus.

Taken together, 84% and 67% of the samples from the feral pigeons and the wood pigeons, respectively, were found to contain a virus with s2m, as summarized in Table 2.

**Table 2:** Results of testing for picornavirus (PiPV-A/PiPV-B), astrovirus (AstV) and coronavirus (CoV) by RT-PCR on samples from feral pigeons (Oslo 2003) and wood pigeons (Akershus 2005).

	Number of positive samples							
Population	PiPV-A	PiPV-B	AstV	CoV*	PiPV-A + PiPV-B	PiPV-A + AstV	PiPV-B + AstV	PiPV + CoV + AstV
**Feral pigeons**								
Juvenile (n = 56)	8	13	53	0	1	8	13	0
Adult (n = 50)	3	9	31	2	0	2	5	1
Unknown (n = 1)	0	0	1	0	0	0	0	0
Virus detection rates	10.3%	20.6%	79.4%	1.9%	0.9%	9.3%	16.8%	0.9%
**Wood pigeons**								
Juvenile (n = 1)	0	0	1	0		0	0	0
Adult (n = 8)	0	0	5	0		0	0	0
Virus detection rates	0.0%	0.0%	66.7%	0.0%		0.0%	0.0%	0.0%

*The CoV results from feral pigeons are taken from an earlier study by Jonassen *et al.*, 2005 [9].

Body weight and results from the necropsy were compared to the findings of different viruses. Most birds appeared healthy, and it was not possible to correlate the presence of a virus to any illness in the birds.

A few samples from each virus family were chosen for further characterization by a 5′ RACE and primer walking strategy, or long-range PCR using conserved motifs of virus families.

### Characterization of novel pigeon picornavirus

We were able to sequence nearly the complete genome from one pigeon picornavirus, PiPV-B, except for the very 5′-end of the 5′UTR. From PiPV-A, 2804 nucleotides were obtained, which covers the complete P3 region and the 3′ UTR. The sequences have been deposited in the GenBank database (http://www.ncbi.nlm.nih.gov/entrez) with accession numbers from FR727144 to FR727145.

**Figure 1:**
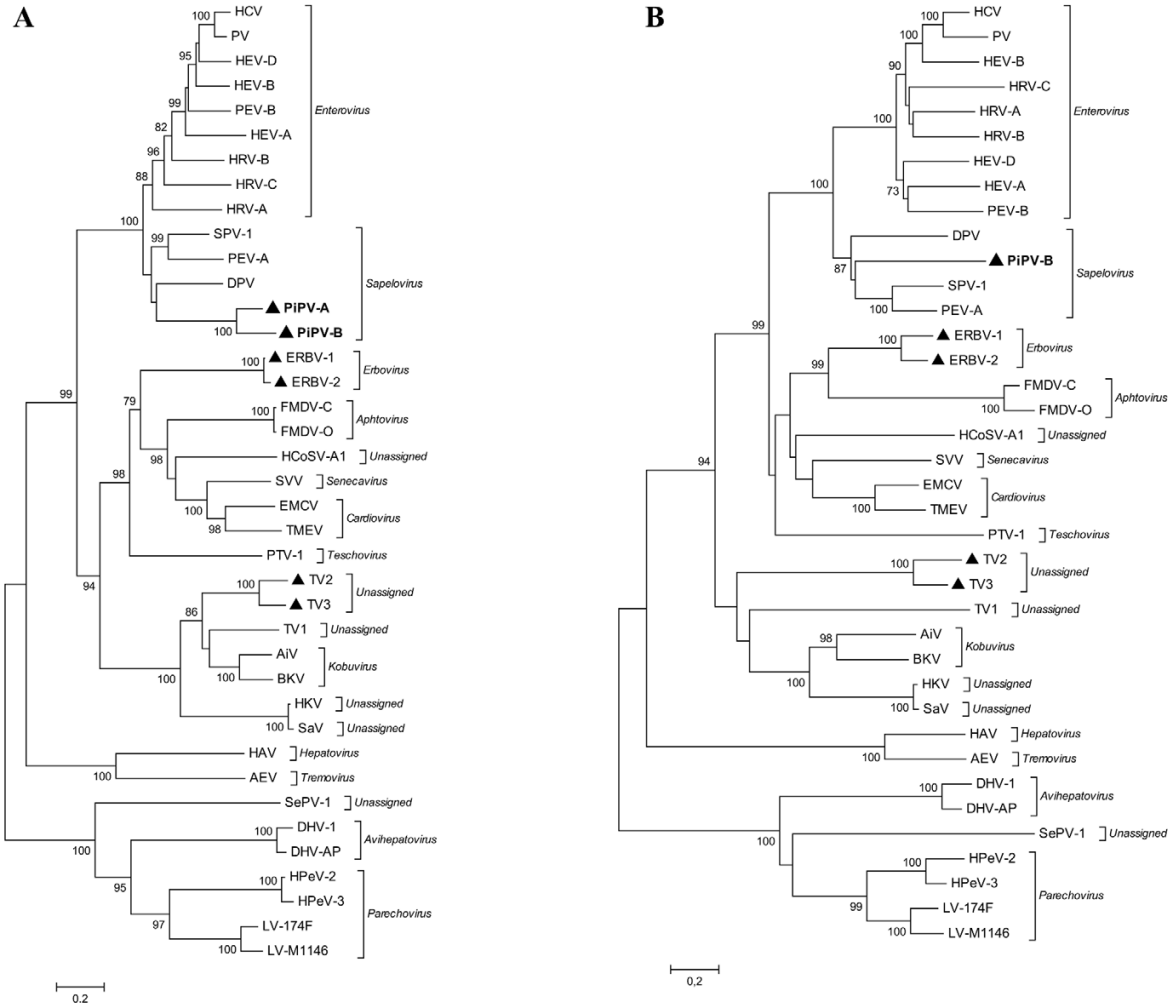
Phylogenetic analysis of A) the complete polymerase protein (3D) and B) the complete P1 capsid region of the picornavirus family. The trees were constructed by the neighbour-joining method using the Jones-Taylor-Thornton matrix-based model of amino acid substitution. Bootstrap values (>70%) from 1000 replicates are shown. Both trees are plotted to the same scale. The novel isolates are indicated by bold lettering. The viruses marked with ▴ harbour s2m.

The sequences were compared to selected picornaviral sequences present in the nucleotide sequence databases. The picornavirus strains that were included in the analysis are listed in Table 3. Phylogenetic trees for picornaviruses were constructed based on the alignment of the putative polymerase protein (3D) and the P1 capsid region (Figure 1). Both phylogenetic analyses show that the new pigeon picornaviruses cluster within the genus *Sapelovirus*.

**Table 3:** Virus strains included in the analyses.

Family/Genus	Species (strain)	Abbreviation	Accession no.
*Picornaviridae*			
Enterovirus	Human enterovirus A	HEV-A	NC_001612
	Human enterovirus B	HEV-B	NC_001472
	Human coxsackievirus A1 (Tompkins)	HCV	AF499635
	Human enterovirus D (70)	HEV-D	NC_001430
	Porcine enterovirus B (UKG/410/73)	PEV-B	NC_004441
	Human poliovirus 1 (Mahoney)	HPV	NC_002058
	Human rhinovirus A (89)	HRV-A	NC_001617
	Human rhinovirus B (70)	HRV-B	DQ473489
	Human rhinovirus C (024)	HRV-C	EF582385
Sapelovirus	Pigeon picornavirus B (03/641)**	PiPV-B	FR727144*
	Pigeon picornavirus A (03/603-7)**	PiPV-A	FR727145*
	Duck picornavirus TW90A	DPV	AY563023
	Simian picornavirus 1 (2383)	SPV-1	NC_004451
	Porcine enterovirus A (V13)	PEV-A	NC_003987
Kobuvirus	Aichi virus (A846/88)	AiV	NC_001918
	Bovine kobuvirus (U-1)	BKV	NC_004421
Teschovirus	Porcine teschovirus 1 (F65)	PTV-1	NC_003985
Erbovirus	Equine rhinitis B virus 1 (P1436/71)	ERBV-1	NC_003983*
	Equine rhinitis B virus 2 (P313/75)	ERBV-2	NC_003077*
Cardiovirus	Encephalomyocarditis virus	EMCV	NC_001479
	Theilovirus (GDVII)	TMEV	NC_001366
Aphtovirus	Foot-and-mouth disease virus C	FMDV-C	NC_002554
	Foot-and-mouth disease virus O	FMDV-O	NC_004004
Hepatovirus	Hepatitis A virus	HAV	NC_001489
Tremovirus	Avian encephalomyelitis virus (Calnek)	AEV	NC_003990
Avihepatovirus	Duck hepatitis virus type 1 (DRL-62)	DHV-1	DQ219396
	Duch hepatitis virus AP (03337)	DHV-AP	DQ256132
Parechovirus	Human parechovirus 2 (Gregory)	HPeV-2	NC_001897
	Human parechovirus 3 (A308/99)	HPeV-3	AB084913
	Ljungan virus (174F)	LV-174F	AF327921
	Ljungan virus (M1146)	LV-M1146	AF538689
Senecavirus	Seneca valley virus (SVV-001)	SVV	DQ641257
Unassigned	Seal picorna virus type 1 (HO.02.21)	SePV-1	EU142040
	Human cosavirus A1 (0553)	HCoSV-A1	NC_012800
	Human klassevirus 1 (02394-01)	HKV	GQ184145
	Salivirus NG-J1	SaV	GQ179640
	Turdivirus 1 (00805)	TV1	GU182407
	Turdivirus 2 (007167)	TV2	GU182409*
	Turdivirus 3 (10878)	TV3	GU182411*
*Astroviridae*			
Mamastrovirus	Human astrovirus 2 (Oxford)	HAstV-2	L13745*
	Human astrovirus 3 (Berlin)	HAstV-3	AF141381*
	Human astrovirus 4 (Guangzhou)	HAstV-4	DQ344027*
	Human astrovirus 8 (Yuk-8)	HAstV-8	AF260508*
	Human astrovirus MLB1 (WD0016)	HAstV-MLB1	FJ402983
	HMO Astrovirus-A (NI-295)	HMOAstV-A	NC_013443*
	HMO Astrovirus-B (NI-196)	HMOAstV-B	GQ415661*
	HMO Astrovirus-C (NI-295)	HMOAstV-C	GQ415662*
	Porcine astrovirus (Tokushima83-74)***	PAstV	AB037272*
	Ovine astrovirus	OAstV	NC_002469*
	Mink astrovirus	MAstV	AY179509*
	Canine astrovirus (3/05)***	CaAstV	FM213330*
	Feline astrovirus (Bristol)***	FAstV	AF056197*
	Bat astrovirus Tm/Guangxi/LD77/2007	BatAstV-LD77	FJ571066*
	Bat astrovirus Tm/Guangxi/LD71/2007	BatAstV-LD71	FJ571067*
	Bat astrovirus Ha/Guangxi/LS11/2007	BatAstV-LS11	FJ571068
	Rat Astrovirus/RS118/HKG/2007	RatAstv-RS118	HM450381
	Bottlenose dolphin astrovirus 1 (Bd1)	BDAstV-1	FJ890355
	California sea lion astrovirus 1 (CSL1)	CslAstV-1	FJ890351*
	California sea lion astrovirus 2 (CSL2)	CslAstV-2	FJ890352*
Avastrovirus	Avian nephritis virus 1 (G-4260)	ANV-1	AB033998*
	Avian nephritis virus 2***	ANV-2	AB046864*
	Turkey astrovirus 1	TAstV-1	Y15936*
	Turkey astrovirus 2	TAstV-2	AF206663
	Duck astrovirus (C-NGB)	DAstV	NC_012437*
	Feral pigeon astrovirus (03/594-6)	FPiAstV-03/594-6	FR727146*
	Feral pigeon astrovirus (03/594-9)	FPiAstV-03/594-9	FR727147*
	Feral pigeon astrovirus (03/603-5)	FPiAstV-03/603-5	FR727148*
	Wood pigeon astrovirus (06/15660-1)	WPiAstV-06/15660-1	FR727149*
*Coronaviridae*			
Gamma-coronavirus	Avian infectious bronchitis virus (Beaudette CK)	IBV	AJ311317*
	Turkey coronavirus (TX-1038/98)	TCoV	GQ427176*
	Duck coronavirus (03/1094)	DCoV	AJ871024*
	Bulbul coronavirus (HKU11-796)	BuCoV	FJ376620*
	Goose coronavirus (03/586-50)	GCoV	AJ871017*
Betacoronavirus	Human SARS coronavirus (Tor2)	SARS CoV	AY274119*
	Bat SARS coronavirus (HKU3-1)	Bat SARS CoV	NC_009694*
	Zaria bat coronavirus (ZBCoV)	ZBCoV	HQ166910*
*Caliciviridae*			
Norovirus	Norovirus dog (Bari/91/07/ITA)	NoV Bari/91	FJ875027*
	Norovirus dog (170/07/ITA)	NoV 170	EU224456*
	Norovirus dog (C33/Viseu/2007/PRT)	NoV C33/Viseu	GQ443611*

*Viruses harbouring s2m.

**Genus assignment suggested in this paper.

***The ORF1b sequence was not available.

The overall genomic organization of the PiPV-B was similar to other picornaviruses. The 7804 nucleotide long genome, excluding the poly(A) tail, was predicted to contain a 618 nucleotide long 5′ UTR (lacking the 5′ terminus), followed by a single long open reading frame, which encodes a 2344 amino acid (aa) polyprotein, and a short 3′ UTR of 160 nucleotides followed by a poly(A) tail. The coding region consists of a leader protein (L), a structural region P1, and the non-structural regions P2 and P3.

Hypothetical polyprotein cleavage sites were determined by the NetPicoRNA server version 1.0 and by alignment of the deduced amino acid sequence with those of sapelo- and enteroviruses. Roughly, cleavage sites should be conserved within a genus. The cleavage sites for PiPV-B are similar to the sapeloviruses. The cleavage sites are all Q/G, except VP4/VP2 which probably is E/G.

#### Coding region

Pairwise percent amino acid identity between PiPV-B and different sapeloviruses and enteroviruses were calculated, and is presented in Table 4, together with the length of each polypeptide/region for PiPV-B and PiPV-A. From the table, it is clear that the new virus sequences are highly divergent from all previously described sapelo- and enteroviruses. The VP2, VP3, VP4, 2C, 3B, 3C and 3D polypeptides of PiPV-B are related most closely to those of sapelovirus, while the other regions differ considerably from those of all known picornaviruses.

**Table 4:** Pairwise percent amino acid identities between the predicted pigeon picornavirus (PiPV-A/PiPV-B) proteins and the corresponding proteins in different sapeloviruses and enteroviruses.

PiPV-B versus:	L	P1	VP1	VP2	VP3	VP4	P2	2A
DPV	NS	36	27	41	40	42	28	NS
SPV-1	NS	37	30	49	43	41	24	NS
PEV-A	NS	39	31	48	44	39	28	NS
HEV-A	NS	33	27	39	34	36	29	NS
HRV-A	NS	30	21	33	36	35	28	NS
Length of PiPV-B	112	840	306	193	227	114	636	191

PiPV-B versus:	2B	2C	P3	3A	3B	3C	3D
DPV	30	43	46	17	45	46	52
SPV-1	22	41	43	22	45	40	49
PEV-A	16	41	42	16	45	40	48
HEV-A	23	39	41	24	41	37	42
HRV-A	17	41	39	20	36	35	47
Length of PiPV-B	115	330	755	92	22	184	457

PiPV-A versus:	P3	3A	3B	3C	3D
PiPV-B	76	63	95	79	77
DPV	46	22	45	45	52
SPV-1	46	23	41	39	53
PEV-A	44	22	45	41	50
HEV-A	41	18	41	37	46
HRV-A	40	15	36	34	49
Length of PiPV-A	754	92	22	184	456

Accession numbers of the sequences used for the alignment and calculation of amino acid identities are listed in Table 3. Lengths of proteins as number of amino acids are noted for PiPV-B (and PiPV-A). NS = no significant alignment.

All sapeloviruses have a leader (L) protein sequence prior to their capsid protein regions. This leader protein is lacking in enteroviruses. In sapelovirus, the leader protein varies in length from 84 amino acids in *Porcine enterovirus A* (PEV-A) [32] to 451 amino acids in *Duck picornavirus* (DPV) [33]. In PiPV-B the leader protein is 112 amino acids. There was little sequence similarity among picornaviral L proteins, and it was not possible to achieve a significant alignment of the PiPV-B L protein with those of other picornaviruses.

Within the sapelovirus genus the 2A polypeptide also varied much in length, from 12 amino acids in DPV to 302 amino acids in *Simian picornavirus 1* (SPV-1) [34]. For PiPV-B the 2A was 191 amino acids, and it shared no similarity with either sapelovirus or enterovirus.

The genomes of most picornaviruses encode two different proteases. Enterovirus encodes two proteases, 2A and 3C, while in aphthovirus, protein L and 3C are proteases. The 2A protein in enterovirus is a trypsin-like protease. It has been shown that the 2A and 3C proteins of PEV-A and SPV-1 both have a GxCG motif, which is the active site of trypsin-like proteases. In DPV, the GxCG motif is located in the L and 3C, but not in the 2A protein [33]. In PiPV-B, the motif is found only in 3C.

The 3D protein is a RNA-dependent RNA polymerase, which is the most conserved picornavirus protein, and it is used to infer phylogenetic relationships among members of the *Picornaviridae* family [35]. This protein in PiPV-B showed 52% amino acid identity to its closest relative, *Duck picornavirus*. Sequences from the two different pigeon picornaviruses, PiPV-A and PiPV-B, showed only 77% amino acid sequence similarity in this region.

#### Noncoding region

The 5′ UTR of PiPV-B consisted of at least 618 nucleotides, but sequence information was incomplete in the 5′ end. Sequence comparisons showed that the 5′ UTR of PiPV-B was most similar to DPV, but only in a short range of the 5′ UTR that corresponds to a part of the internal ribosome entry site (IRES) element (domain III_d_ to III_f_) (68.9% identity in 106 nucleotides). There were no similarities found outside the IRES element.

**Figure 2:**
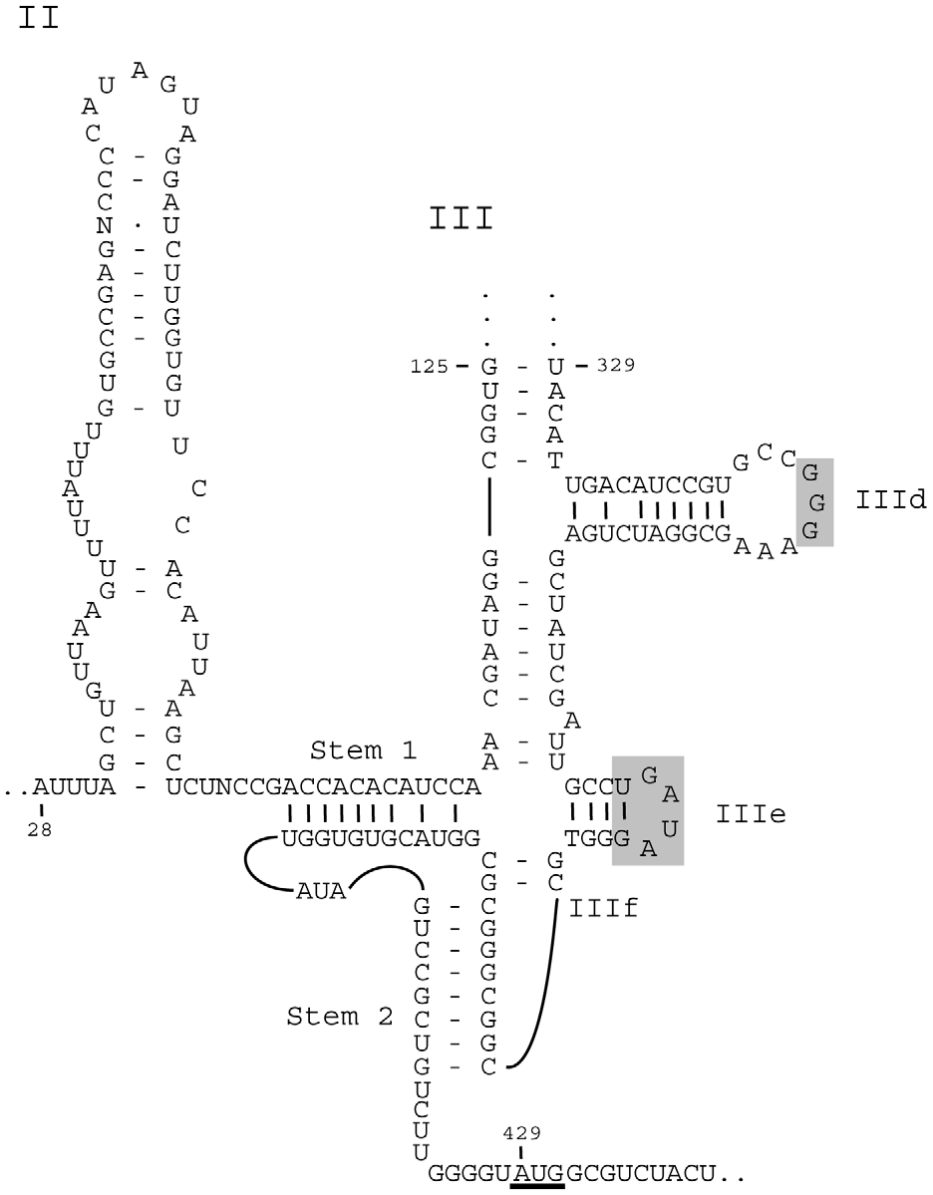
Model of the predicted secondary structure of PiPV-B IRES. Domains are labelled II and III, stem-loops are labelled IIId, IIIe etc. Stem 1 and stem 2 are elements of the pseudoknot. The underlined AUG triplet in position 429 is the first AUG codon after the pseudoknot. Shaded rectangles indicate bases in domain III that are conserved in the IRES type IV elements. The predicted secondary structure upstream of stem-loop IIId (the sequence between nucleotide 125 and 329) is not shown. The structure was predicted using Mfold.

Analysis of RNA secondary structure by the Mfold program showed that the IRES structure of PiPV-B was most similar to type IV IRES elements (Figure 2). The IRES of PiPV-B lacked the polypyrimidine tract located 25 nucleotides upstream of the AUG start codon in picornavirus IRES types I-III, and harboured the other IRES type IV signature elements, i.e. a shorter length and the presence of two domains, termed domain II and III, where domain III includes a number of distinct stem-loops, e.g. III_d_ (consisting of 20–28 nucleotides and containing a strictly conserved GGG motif within the terminal loop), III_e_ (highly conserved and consisting of 12 nucleotides) and a pseudoknot, III_f_. Domain II in PiPV-B consisted of 62 nucleotides, and lacked the GAA and AGUA motifs present in almost all IRES type IV elements, except for PEV A, while domain III consisted of about 275 nucleotides [36]–[38].

**Figure 3:**
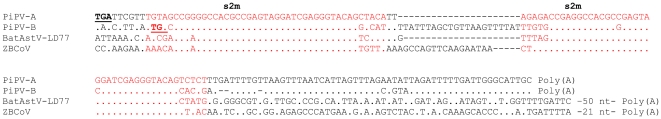
Alignment of the genomic 3′ –end of the two subtypes of the novel pigeon picornavirus, the astrovirus BatAstV-LD77 and the coronavirus ZBCoV. The two adjacent s2m are shown in red. Stop codon of the polyprotein is underlined (part of the first s2m for PiPV-B). Dots indicate nucleotides identical to the PiPV-A, gaps are shown as dashes.

The 3′ UTRs of picornaviruses are generally quite short, ranging from 34 nucleotides in *Seal picornavirus type 1* [39] to 314 nucleotides in *Duck hepatitis virus type 1* [33]. The 3′ UTR was 152 nucleotides long in PiPV-A, and 160 nucleotides long in PiPV-B. They shared 70% identity at the nucleotide level, but they had no significant similarity with any other picornaviruses, except for s2m in the equine rhinitis B viruses. Both PiPV-A and PiPV-B harboured two adjacent s2m, called (s2m)_2_, in their 3′ UTR. Respectively, there were 2 and 24 nucleotides between the two adjacent s2m, in PiPV-A and PiPV-B (Figure 3).

### Characterization of astrovirus

Three astroviruses from feral pigeon and one from wood pigeon were selected for further characterization. From each genomic RNA about 3200 nucleotides, which partially covers the polymerase gene (about 300 amino acids in the C terminal part of ORF1b), the complete capsid gene (ORF2) and the 3′ UTR, were obtained. The sequences have been deposited in the GenBank database (http://www.ncbi.nlm.nih.gov/entrez) with accession numbers from FR727146 to FR727149.

The sequences were compared to selected astrovirus sequences present in the nucleotide sequence databases, shown in Table 3. Pairwise comparisons based on amino acid sequences of the partial ORF1b and the complete ORF2 were performed to determine the relationship of the feral and wood pigeon astroviruses with other astroviruses. The astrovirus isolates from feral pigeon shared 70–71% amino acid identity in ORF2 with each other, whereas the amino acid identity between feral pigeon and the wood pigeon astrovirus was of 54–56%. Higher identity was detected for the partial sequence related to ORF1b, being of 97–98% among feral pigeon astrovirus and 96% between feral pigeon and wood pigeon astrovirus. We found that both feral pigeon astroviruses and wood pigeon astrovirus were more closely related to the avian nephritis viruses (ANV) than to other astroviruses (86–87% amino acid identity in ORF1b).

**Figure 4:**
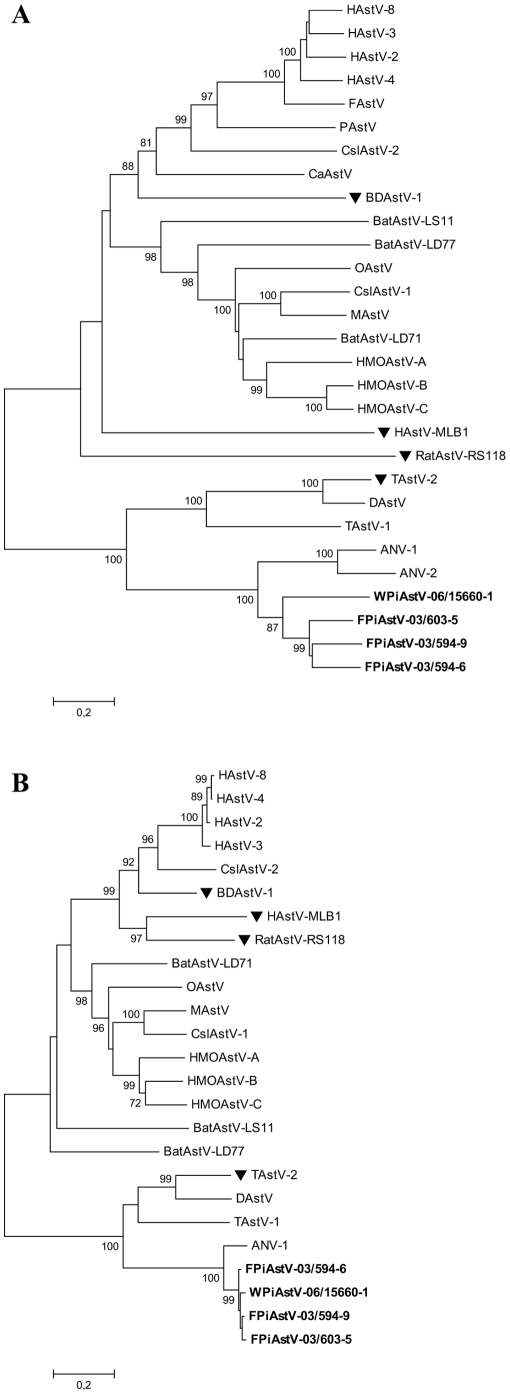
Phylogenetic analysis of A) the complete capsid protein (ORF2) and B) part of the polymerase protein (about 300 amino acids in the C-terminal part of ORF1b) of the astrovirus family. The trees were constructed by the neighbour-joining method using the Jones-Taylor-Thornton matrix-based model of amino acid substitution. Bootstrap values (>70%) from 1000 replicates are shown. Both trees are plotted to the same scale. The novel isolates are indicated by bold lettering. The viruses marked with ▾ do not harbour s2m.

To gain further insight into the relationship of the pigeon astroviruses with other astroviruses, phylogenetic trees for astrovirus were constructed based on the alignment of the putative amino acid sequences encoded by ORF1b and ORF2 (Figure 4). The phylogenetic analyses of ORF2 (Figure 4A) show that the isolates from feral pigeons cluster together to form a new group of viruses within the avian genogroup. The isolate from wood pigeon grouped separately from the feral pigeon group. The novel astroviruses were most closely related to ANV, although they were highly divergent from them (42–49% identity at the amino acid level). Similar tree topology was observed when ORF1b was analysed (Figure 4B). Sequence information in that part of the genome was not available for porcine astrovirus, canine astrovirus, feline astrovirus or ANV-2.

**Figure 5:**
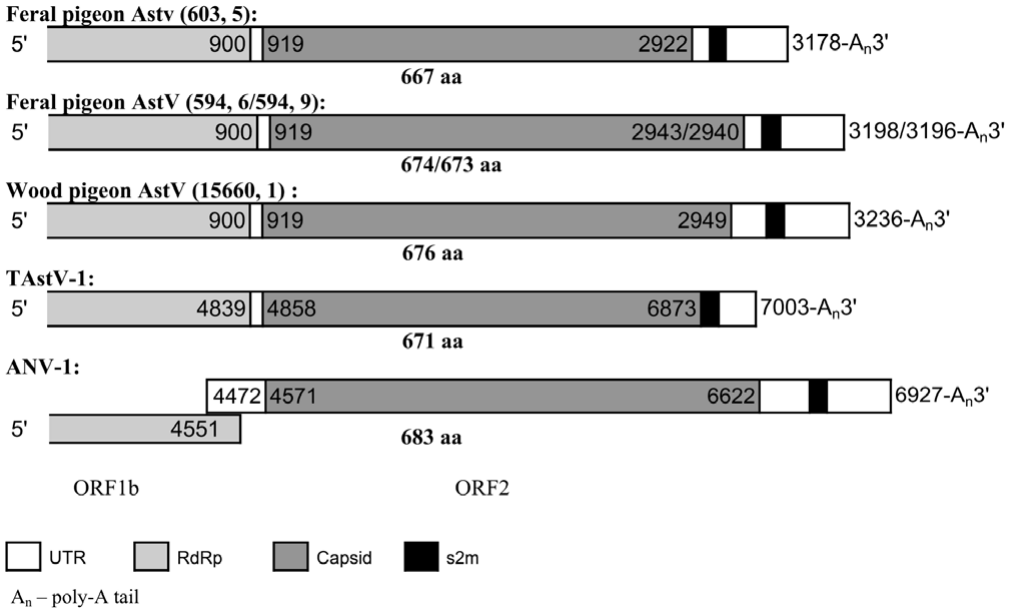
Diagram of the organization of the 3′ part of the avastrovirus genomes. The nucleotide positions of the start and stop of each open reading frame are shown relative to the beginning of the genome for TAstV-1 and ANV-1. Feral pigeon AstV (594, 6/594, 9) represent two different virus strains.

Both feral and wood pigeon astroviruses have the same genomic organization as the turkey astroviruses, with ORF1b and ORF2 in the same reading frame, as shown in Figure 5. In contrast, the ORF2 of *ANV-1*, *Duck astrovirus* and most mammalian astroviruses (except *Mink astrovirus*) are not in the same reading frame as ORF1b [2], [40]–[43].

The predicted capsid protein of pigeon astrovirus encoded by ORF2 has a length of 667–676 amino acids, which is among the smallest astrovirus capsid protein known. Capsid proteins of astrovirus infecting different hosts are highly divergent [44]. The N-terminal half of the ORF2 protein, which is more conserved than the C-terminal half and proposed to constitute the assembly domain of the viral capsid [44]–[46], was also found to be relatively conserved in the pigeon astroviruses.

In astrovirus, the 3′ UTR range from 58 nucleotides in *Human astrovirus MLB1* to 305 nucleotides in ANV-1. The pigeon astroviruses contains a 3′ UTR of 255–287 nucleotides, where the s2m motif is found, followed by a poly(A) tail.

## Discussion

In this paper we report the discovery of novel picornaviruses and astroviruses in feral and wood pigeons, and identified viruses in yet another family with a tandem copy of the s2m mobile motif.

The novel pigeon picornaviruses (PiPV) were found in 30% of the pigeons tested, suggesting that these viruses are relatively common among pigeons.

The almost complete genome sequence of PiPV-B was shown to have a genome organization and several features typical of sapeloviruses, including a putative leader protein encoded upstream of the structural proteins (in the polyprotein N-terminal) and an IRES element that is highly related to the IRES type IV in sequence and overall secondary structure [36]. Even if there is a relatively low sequence conservation within the pseudoknot (III_f_) between the IRES type IV elements, the pseudoknot has a highly conserved structure [47], [48]. Due to a relatively long domain III, the IRES element of PiPV-B was considerably larger (about 400 nucleotides) than the other IRES group IV elements, except for the IRES element of SPV-1 which is approximately 440 nucleotides [47].

IRES type IV elements have been subdivided into three groups on the basis of length and sequence covariation of the pseudoknot and adjacent elements. Sapeloviruses belong to group B, together with Porcine teschovirus 1 and Duck hepatitis virus type 1 [37]. Sequence comparisons between PiPV-B and the viruses in group B, showed that PiPV-B also belong to this group.

For PiPV-B, it could seem that the first AUG codon downstream of the pseudoknot was silent, if the start of its polyprotein was homologous to the start of sapelovirus polyproteins, and that the functional initiation codon thus was located 187 nucleotides downstream of this pseudoknot, with four AUG codons in between. This is quite common for IRES elements, but further analysis should be performed to confirm the N terminal part of the polyprotein.

In addition, phylogeny and comparisons with other picornaviruses showed that overall the PiPV-B polypeptides were most closely related to those of the sapeloviruses, sharing 52% amino acid sequence identity with *Duck picornavirus* in the 3D region.

The novel pigeon picornaviruses did not seem to be associated with any disease or growth inhibition in the pigeons, which is a property shared with all the sapeloviruses identified so far, none of which being particularly pathogenic for their hosts [33].

Although PiPV-B is most closely related to members of the genus Sapelovirus, PiPV-B are clearly distinct from DPV, SPV-1 and PEV-A. According to the International Committee on Taxonomy of Viruses (ICTV) Picornaviridae Study Group (PSG) (www.picornastudygroup.com), the Leader, 2A, 2B and 3A polypeptides would normally be expected to be homologous between members of a genus. The amino acid sequences of these polypeptides in PiPV-B share none or very little sequence similarity with the corresponding polypeptides in sapeloviruses. DPV, PEV-A and SPV-1 also have very divergent 2A, 2B and 3A polypeptides. In FMDV and human rhinoviruses, the 2B and 3A proteins have been shown to be involved in host-cell tropism and virulence [49], [50]. The broad range of hosts infected by the sapelovirus genus could be an explanation for why these proteins differ so much.

Also according to the PSG, members of a genus should normally share >40%, >40% and >50% amino acid identity in P1, P2 and P3 genome regions respectively. PiPV-B shared only 39% amino acid identity in the P1 region, 28% amino acid identity in the P2 region and 46% amino acid identity in the P3 region with its closest relative in the sapelovirus genus, and the classification of the pigeon picornaviruses is therefore currently uncertain.

PiPV-A shared only 77% amino acid identity with PiPV-B in the 3D region, suggesting that PiPV-A and PiPV-B should be classified as two different species. However, more sequence information from PiPV-A is needed to draw any conclusions about this.

A very high prevalence of astrovirus was found among feral pigeons and wood pigeons in the present study. Astrovirus was detected in almost all juvenile birds, while the detection rate among the adults was 62%. Recently, astrovirus detection rates up to 100% have been reported in insectivorous bats in Hong Kong [41]. Both ANV and TAstV have also been shown to be widely distributed [40], [51]. In contrast, in other species such as dogs or cats, the detection of astrovirus appears very sporadic [52], but astrovirus is often isolated together with other enteric pathogens, both in animals and in humans [53]. In mammals, astrovirus causes gastroenteritis, while in birds, astroviruses are associated with a broader spectrum of diseases, such as enteritis, hepatitis and nephritis. We were not able to detect any correlation between the presence of astrovirus and illness in the birds.

The findings show that the identified feral pigeon astrovirus is a novel avastrovirus clearly distinct from other astroviruses. It has <87% and <44% amino acid identity to other known astroviruses in the ORF1b and ORF2 regions, respectively. Its closest relative is ANV-1. Phylogenetic analyses of ORF2 show that the wood pigeon astrovirus grouped separately from the feral pigeon astroviruses (54–56% identity at the amino acid level), but in ORF1b they grouped together.

According to ICTVdB - The Universal Virus Database, version 4 (http://www.ncbi.nlm.nih.gov/ICTVdb/ICTVdB/), species are defined on the basis of host of origin; we therefore suggest two novel species of astrovirus, *Feral pigeon astrovirus* and *Wood pigeon astrovirus*, to be included in the genus Avastrovirus.

The three astrovirus isolates from feral pigeon which were characterized were genetically diverse, although the host pigeons were captured in the same area on two subsequent days. It has previously been reported that the amino acid sequence identities of the RdRp gene between four groups of avastrovirus, i.e. TAstV-1, TAstV-2, ANV and chicken-origin astroviruses, detected in different regions also were highly diverse, ranging from 50,3% to 73,8% identity. There were also multiple phylogenetic subgroups within each group. Phylogenetic analysis revealed no clear assortment by geographic region or isolation date [51], [54]. The distances between both the polymerase and the capsid sequences of the feral pigeons were comparable to the distances between the HAstV-1 to HAstV-8 types. The sequence identity of the entire capsid protein precursors between different serotypes of HAstVs was 66–76% at the nucleotide level [46], while the sequence identity between the feral pigeons at the nucleotide level was 66–68% in the same region, suggesting a variety of astrovirus genogroups within feral pigeons, analogous to HAstV genogroups.

By using s2m-specific amplification, we could identify both a novel picornavirus and a novel astrovirus. In addition, a novel coronavirus had earlier been identified and characterized with this method in the same bird species [9], and it is likely that other animal species, including humans, may carry not yet identified s2m harbouring viruses. Several of the viruses carrying s2m are important animal pathogens, and the conserved nature of the motif might make it an adequate target for antivirals.

s2m has so far only been identified in viruses with a poly(A)-tailed genome, and always located within 200 nucleotides from the poly(A) tail. In the novel pigeon astroviruses, s2m was located entirely within the 3′ UTR (Figure 5), similarly to the location of s2m in all avastroviruses, except for *Duck astrovirus* (DAstV), where s2m is partially located in the capsid region and partially in the 3′ UTR, as in mamastrorviruses. S2m in picorna- and coronaviruses is located entirely within the 3′ UTR, while s2m in norovirus is located partially in the minor capsid complex (ORF3) and partially in the 3′ UTR. The invariant positioning of s2m close to the 3′ end of the positive RNA strand may suggest that its function is connected to the replication or the stability of the viral RNA molecule. The wide range of viruses harbouring s2m suggests that its function is not by interaction with virus proteins. Even considering the wide range of host cell types, it might have a function based on interaction with conserved cellular biomolecules. The viruses harbouring s2m have different strategies for translation initiation, but a role of s2m in viral RNA translation through binding to one or more proteins possessing an oligomer-binding-like fold has been proposed based on tertiary structural comparisons [17]. Three lines of evidence suggest that a mechanism for facilitation of its horizontal transfer is imbedded in the structure itself: 1) the relative high number of recombination or excision events necessary to explain its distribution among virus groups, 2) the wide range of RNA primary and secondary structures surrounding the motif and 3) that the excision or insertion of s2m seem to be restricted to the structure without surrounding sequences.

The novel picornaviruses were most related to the genus *Sapelovirus*, and not to the other s2m-harbouring picornaviruses; the equine rhinitis B viruses and wild bird turdiviruses. This suggests acquisition of s2m in these picornavirus groups in separate recombination events, as proposed for the SARS coronavirus and avian coronavirus [55]. The novel picornaviruses were all found in the cloacal swabs of the sampled animals. The high prevalence of viruses harbouring s2m and multiple RNA viruses in cloacal swabs from pigeons suggested a favourable condition for recombination between unrelated viruses, and one of the pigeons was actually simultaneously shedding 3 different s2m-harbouring viruses (corona-, picorna- and astrovirus).

**Figure 6:**
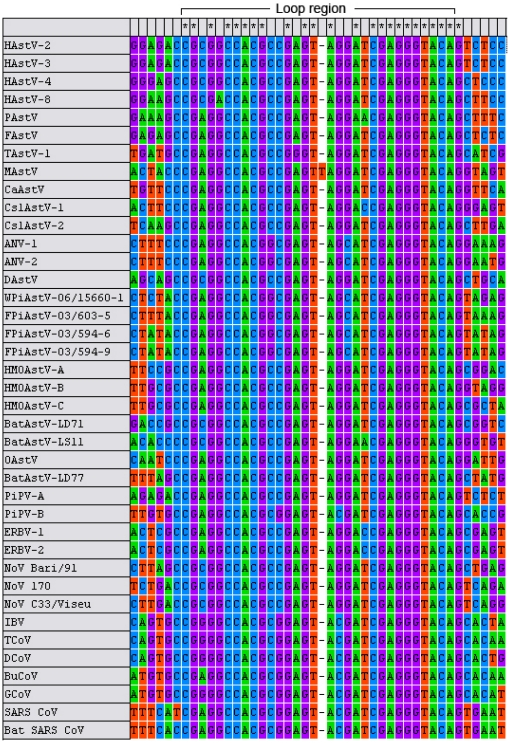
Nucleotide alignment of s2m from various astroviruses (AstV), picornaviruses (PV), coronaviruses (CoV) and noroviruses (NoV) species. The 24 nucleotides that are absolutely invariant, are marked by asterisks above the sequences.

Figure 6 shows the primary structure of s2m. 71% of the sequence in the loop region (31 nucleotides) is invariable. Differences in the stem are usually compensated by other differences, to maintain complementarity. These compensating substitutions indicate that the RNA secondary structure is important for the function and that the stem is biologically important.

There is little sequence similarity in the regions flanking s2m, suggesting that only s2m has been transferred between the different virus species. This implies that a replication-dependent recombination must have involved two consecutive template switches. This also implies that if replication-dependent recombinations have taken place, the mechanisms were probably not homologous recombination. While replication-dependent recombination is the main mechanism of RNA recombination observed in animal viruses, recombination by cleavage and ligation, which is reported to occur in bacteriophage Qβ, poliovirus and pestivirus [56]–[58], can not be completely ruled out. It is possible that s2m has been transferred via a replication independent mechanism [1].

Since both novel picornaviruses harboured two adjacent s2m, called (s2m)_2_, this could suggest a possible increased functional effect of this motif, when present in two copies. (s2m)_2_ has previously been observed in one bat astrovirus, BatAstV-LD77, and more recently in one bat coronavirus, ZBCoV [6], [59] (Figure 3), suggesting a selective advantage for tandem copy of this mobile element in several virus species. S2m is apparently easily transferred from one virus to another, and is so far the only evidenced mobile element of RNA virus genomes, of yet not ascertained function.
